# Blood Pressure Changes Following Antihypertensive Medication Reduction, by Drug Class and Dose Chosen for Withdrawal: Exploratory Analysis of Data From the OPTiMISE Trial

**DOI:** 10.3389/fphar.2021.619088

**Published:** 2021-04-20

**Authors:** James P. Sheppard, Mark Lown, Jenni Burt, Gary A. Ford, F. D. Richard Hobbs, Paul Little, Jonathan Mant, Rupert A. Payne, Richard J. McManus, On behalf of the OPTiMISE Investigators

**Affiliations:** ^1^ Nuffield Department of Primary Care Health Sciences, University of Oxford, Oxford, United Kingdom; ^2^ Primary Care Research Group, University of Southampton, Southampton, United Kingdom; ^3^ The Healthcare Improvement Studies Institute, University of Cambridge, Cambridge, United Kingdom; ^4^ Radcliffe Department of Medicine, University of Oxford, and Oxford University Hospitals NHS Foundation Trust, Oxford, United Kingdom; ^5^ Primary Care Unit, Department of Public Health and Primary Care, University of Cambridge, Cambridge, United Kingdom; ^6^ Centre for Academic Primary Care, Population Health Sciences, University of Bristol, Bristol, United Kingdom

**Keywords:** deprescribing, older adults, hypertension, polypharmacy, Multi-morbidity, beta-blockers, calcium channel blockers, defined daily dose

## Abstract

**Aims:** Deprescribing of antihypertensive drugs is recommended for some older patients with polypharmacy, but there is little evidence to inform which drug (or dose) should be withdrawn. This study used data from the OPTiMISE trial to examine whether short-term outcomes of deprescribing vary by drug class and dose of medication withdrawn.

**Methods:** The OPTiMISE trial included patients aged ≥80 years with controlled systolic blood pressure (SBP; <150 mmHg), receiving ≥2 antihypertensive medications. This study compared SBP control, mean change in SBP and frequency of adverse events after 12 weeks in participants stopping one medication vs. usual care, by drug class and equivalent dose of medication withdrawn. Equivalent dose was determined according to the defined daily dose (DDD) of each medication type. Drugs prescribed below the DDD were classed as low dose and those prescribed at ≥DDD were described as higher dose. Outcomes were examined by generalized linear mixed effects models.

**Results:** A total of 569 participants were randomized, aged 85 ± 3 years with controlled blood pressure (mean 130/69 mmHg). Within patients prescribed calcium channel blockers, higher dose medications were more commonly selected for withdrawal (90 vs. 10%). In those prescribed beta-blockers, low dose medications were more commonly chosen (87 vs. 13%). Withdrawal of calcium channel blockers was associated with an increase in SBP (5 mmHg, 95%CI 0–10 mmHg) and reduced SBP control (adjusted RR 0.89, 95%CI 0.80–0.998) compared to usual care. In contrast, withdrawal of beta-blockers was associated with no change in SBP (−4 mmHg, 95%CI −10 to 2 mmHg) and no difference in SBP control (adjusted RR 1.15, 95%CI 0.96–1.37). Similarly, withdrawal of higher dose medications was associated with an increase in SBP but no change in BP control. Withdrawal of lower dose medications was not associated with a difference in SBP or SBP control. There was no association between withdrawal of specific drug classes and adverse events.

**Conclusion:** These exploratory data suggest withdrawal of higher dose calcium channel blockers should be avoided if the goal is to maintain BP control. However, low dose beta-blockers may be removed with little impact on blood pressure over 12-weeks of follow-up. Larger studies are needed to confirm these associations.

## Introduction

Antihypertensive treatment is effective at preventing stroke and cardiovascular disease in older high-risk patients with hypertension (Beckett et al., 2008; SPRINT Investigators et al., 2015; Thomopoulos et al., 2018) and many individuals aged 80 years or older are prescribed therapy (Sheppard et al., 2012). Such patients are also more likely to live with multiple long-term conditions (Barnett et al., 2012) leading to polypharmacy, which increases an individual’s likelihood of hospitalization due to adverse events (Pirmohamed et al., 2004; Sato and Akazawa, 2013). It is unclear whether intensive blood pressure lowering is safe and effective in older patients with multi-morbidity and frailty. Previous trials have found that frailty has no modifying effect on the efficacy of blood pressure lowering in older patients (Warwick et al., 2015; Williamson et al., 2016), however, such trials may not have included very frail patients seen in the general population (Sheppard et al., 2020a; Sheppard et al., 2020b). In contrast, evidence from meta-analyses of randomized controlled trials (Bejan-Angoulvant et al., 2010; Thomopoulos et al., 2016) and observational studies (Tinetti et al., 2014; Benetos et al., 2015; Mansfield et al., 2016) suggests that aggressive lowering of systolic blood pressure (i.e. to less than 130 mm Hg) and multiple antihypertensive prescriptions may be harmful, particularly in older patients with polypharmacy and multi-morbidity (Tinetti et al., 2014; Thomopoulos et al., 2016).

Guidelines therefore recommend using clinical judgment when prescribing in frail older patients (National Heart Foundation of Australia, 2016; National Guideline Centre, 2019; Liu et al., 2020), emphasizing a personalized approach to care which might include attempts to improve quality of life through deprescribing (Benetos et al., 2016; National Guideline Centre, 2016). The Optimizing Treatment for MIld Systolic hypertension in the Elderly (OPTiMISE) trial (Sheppard et al., 2020c) examined a structured approach to antihypertensive medication reduction in older patients with multi-morbidity and controlled systolic hypertension, prescribed two or more antihypertensives. The overarching aim of the OPTiMISE trial was to reduce polypharmacy without blood pressure becoming uncontrolled. The trial showed that a strategy of medication reduction results in similar proportions of patients with controlled systolic blood pressure (<150 mm Hg) at 12 weeks when compared to continuing antihypertensives. No differences were observed in serious adverse events or quality of life, although systolic/diastolic blood pressure did increase modestly by 3/2 mm Hg in the medication reduction group (Sheppard et al., 2020c).

**FIGURE 1:**
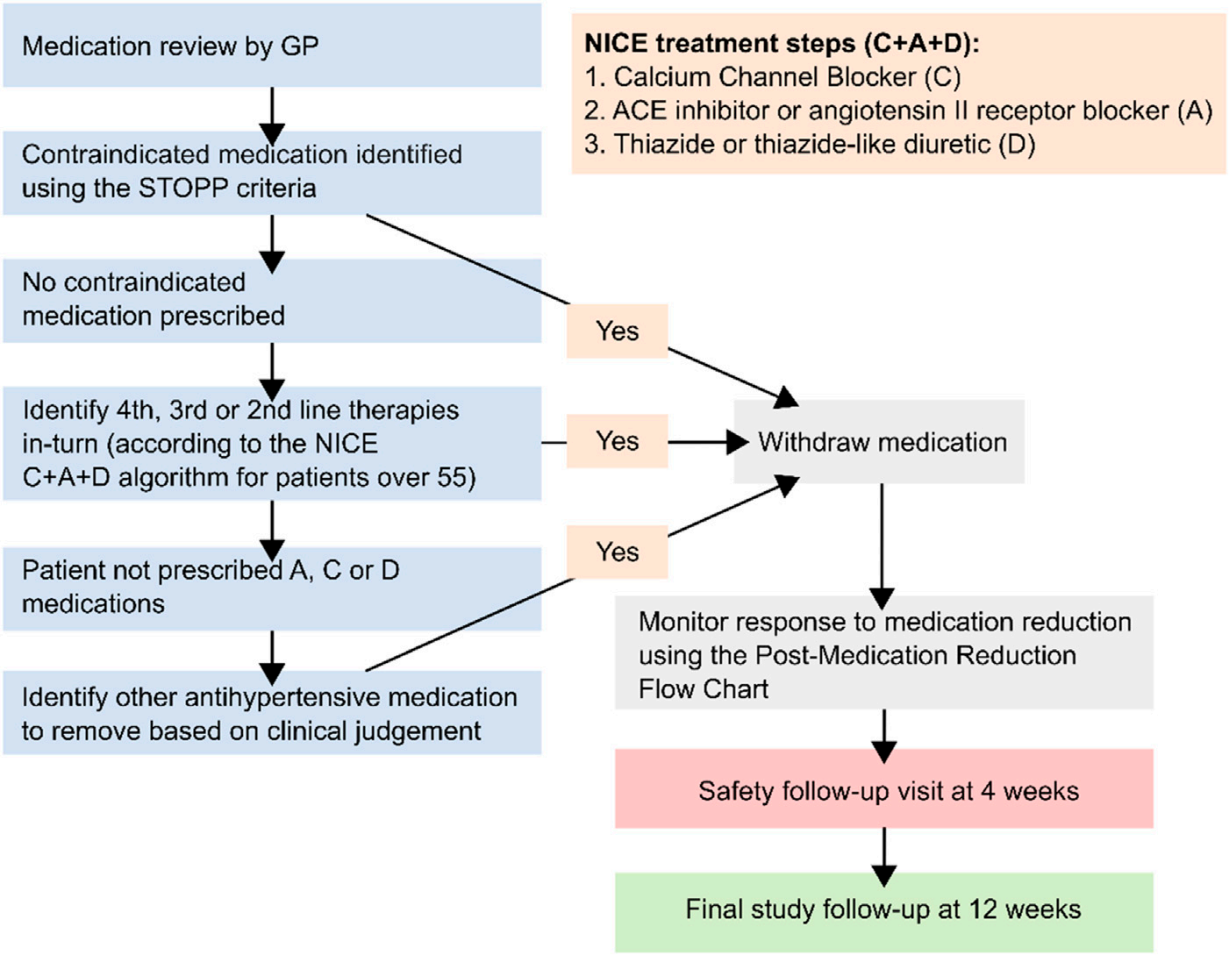
Medication reduction algorithm given to general practitioners participating in the Optimize trial NICE = National Institute for Health and Care Excellence. Contraindicated medications described in the STOPP START criteria (Gallagher et al., 2008). Figure adapted from previous publications about this trial (Sheppard et al., 2018; Sheppard et al., 2020c).

There is little evidence to guide antihypertensive deprescribing (Krishnaswami et al., 2019), and therefore physicians participating in the trial were instructed to decide which antihypertensive should be removed based on advice from a medication reduction algorithm (Figure 1). The present study aimed to examine whether this choice was associated with blood pressure changes and adverse events in the trial.

## Methods

### Design

This was a post-hoc exploratory analysis of data from the OPTiMISE trial of antihypertensive medication reduction (Sheppard et al., 2020c). All participants randomized in the trial, who did not withdraw consent, were included in the analysis. The trial was approved by an NHS Research Ethics Committee (South Central - Oxford A; ref 16/SC/0628) and the Medicines and Healthcare products Regulatory Agency (MHRA; ref 21,584/0371/001-0001). All participants gave written informed consent. Details of patient recruitment and data collection are described in detail elsewhere (Sheppard et al., 2018; Sheppard et al., 2020c).

### Study Population

Individuals were eligible if they were aged ≥80 years, with systolic blood pressure at baseline <150 mm Hg (based on the mean of the second and third readings taken, after 5 min of rest) and prescribed two or more antihypertensive treatments for at least 12 months. Recruiting primary care physicians were asked to only enroll patients whom in their opinion might potentially benefit from medication reduction due to existing polypharmacy, co-morbidity, non-adherence or dislike of medicines, and/or frailty. This clinical judgment was considered important given the current lack of evidence as to who should be targeted for such an intervention. Patients with a history of heart failure due to left ventricular dysfunction or myocardial infarction/stroke in the preceding 12 months, secondary hypertension or lacking in capacity to consent were excluded.

Potentially eligible patients were identified from searches of electronic health records in participating sites and sent letters of invitation. Those expressing an interest attended a screening appointment.

### Randomization and Blinding

Participants were allocated (1:1 allocation ratio) to one of the two study groups using a non-deterministic minimization algorithm, with minimization designed to balance site and baseline systolic blood pressure, via a fully validated, web-based, password protected system. Investigators and participants were unaware of the treatment allocation prior to consent and baseline assessments. The trial used an unblinded design with patients and investigators not masked to randomization group.

### Medication Reduction Intervention

Participating primary care physicians reviewed each participant’s medication regimen before randomization and decided which antihypertensive would be removed if they were allocated to medication reduction, using a pre-specified algorithm (Figure 1). This algorithm recommended reducing medications in reverse of the C + A + D NICE treatment algorithm. Following an adverse event possibly related to abrupt discontinuation of a beta-blocker, gradual withdrawal of these medications was encouraged to avoid rebound adrenergic hypersensitivity. For individuals randomized to medication reduction, physicians were asked to monitor blood pressure at a 4 week follow-up visit and reinstate treatment if it consistently rose above 150 (systolic) or 90 (diastolic) mm Hg, or in the case of adverse events or accelerated hypertension. Patients in the control group were given usual care and no medication changes were mandated.

### Outcomes

Outcomes examined in this analysis were not pre-specified before the end of the trial and should be treated as exploratory. Outcomes included between group differences in systolic blood pressure control, adverse events and change in systolic and diastolic blood pressure at follow-up by drug class and dose of medication chosen for withdrawal. Adverse events were defined as any clinical event occurring during follow-up, regardless of whether it was deemed to be possibly, probably or definitely related to the intervention by the treating physician. Systolic and diastolic blood pressure were defined as the mean of the second and third consecutive readings taken at 1 min intervals. Measurements were taken in the seated position, using the clinically validated BpTRU blood pressure monitor (Mattu et al., 2004) after a period of 5 min of rest.

### Definition of Subgroups

For each analysis by drug class, groups were determined according to drug classifications in the British National Formulary (BNF) (Royal Pharmaceutical Society, 2020). Equivalent dose of medication was determined by converting the doses of each drug chosen for withdrawal into a common unit of measure using the World Health Organisation (WHO) defined daily dose (DDD) for each medication type (World Health Organisation (WHO) Collaborating Centre for Drug Statistics Methodology, 2020). For example, the DDD for Ramipril is 2.5 mg (World Health Organisation (WHO) Collaborating Centre for Drug Statistics Methodology, 2020), so if a drug was prescribed at 1.25 mg, it would be classified in the present analysis as having a medication equivalent dose of 0.5. For the purposes of these analyses, participants were divided into two groups according to the equivalent dose of medication chosen for withdrawal; low dose medications were those prescribed at less than the DDD (i.e. an equivalent medication dose of <1). Higher dose medications were those prescribed at the DDD or higher doses (i.e. an equivalent medication dose of ≥1).

### Covariates

Data relating to participant demographics, body mass index, blood pressure, cognition (Montreal Cognitive Assessment [MoCA] Score) (Nasreddine et al., 2005), functional independence (modified Rankin score) (Sulter et al., 1999), frailty (electronic/Searle Frailty Index) (Searle et al., 2008; Clegg et al., 2016), past medical history and treatment prescriptions were collected at baseline via participant questionnaires and review of the electronic health record. Predictors of physician drug choice were selected to reflect trial guidance provided on medication reduction. This included the number of pre-existing medication prescriptions, concurrent morbidities, frailty (defined using the electronic frailty index) (Clegg et al., 2016), age, sex and systolic blood pressure at baseline. Multivariate models examining the association between medication withdrawal and outcomes were adjusted for factors found to be predictive of medication choice for withdrawal and missing follow-up data, including baseline systolic blood pressure, gender, MoCA score (Nasreddine et al., 2005), EQ-5D-5L Index (Herdman et al., 2011), Searle Frailty Index (Searle et al., 2008) and primary care site.

### Statistical Analysis

Descriptive statistics were used to describe the study population, the proportion of participants maintaining medication reduction and the proportion experiencing no increase in systolic blood pressure in the intervention group at follow-up. These were estimated by drug class and dose of medication chosen for withdrawal. Since the choice of drug to withdraw was not fixed, but rather at the discretion for the treating physician, multivariable logistic regression was used to examine predictors of physician drug choice. Statistically significant predictors were included as factors for adjustment in the main analysis.

Data from participants examining outcomes of medication reduction by drug class and medication dose were analyzed according to the groups to which they were allocated (i.e. by intention to treat). The relative risk (RR) for blood pressure control and adverse events between groups were examined by drug class and medication dose chosen for withdrawal using a robust Poisson regression model. Each model was adjusted for baseline systolic blood pressure, covariates predictive of drug choice for medication withdrawal and those predictive of missing blood pressure data at follow-up (identified in the preparatory analyses). Since the treating physician’s choice of medication to withdraw was made prior to consent and randomization, data were available for all randomized participants, even though only half went on to have the medication withdrawn. Therefore, models compared patients withdrawing specific drugs (the intervention group) to patients where the same drug was selected for withdrawal, but treatment was actually continued (usual care). Separate models were fitted according to the drug class and medication dose chosen for withdrawal. Adjusted mean difference in change in blood pressure was analyzed by means of generalized linear mixed model with binomial error and log link, with factors predictive of physician choice of drug to withdraw and baseline systolic blood pressure, gender, cognitive function (MoCA Score), EQ-5D-5L Index and Searle Frailty Index as fixed effects and primary care site as a random effect.

All data were analyzed using Stata statistical software (version 16.0, College Station TSL, StataCorp, 2019). Data are presented as means, medians and proportions with 95% confidence intervals (CI) unless otherwise stated.

## Results

A total 569 patients were recruited to the trial from 69 general practices in Central, Eastern and Southern England. The characteristics of participants in the trial were broadly comparable to those of a similar age group in the general population (Supplementary Table S1). Two hundred and eighty-two participants (49.6%) were randomized to the medication reduction intervention and 287 participants (50.4%) were randomized to usual care. A total of 534 (93.8%) participants attended 12-week follow-up and provided valid blood pressure readings. Participants were well matched for all variables at baseline, with a mean age of 85 years, multi-morbidity (mean 5.8 morbidities; 98.4% participants had ≥2 morbidities including hypertension) and polypharmacy (median four medications; Table 1). Mean blood pressure at baseline was 130/69 mm Hg and individuals were taking a median of 2 (IQR 2–3) antihypertensive medications.

**TABLE 1:** Baseline demographics and clinical characteristics.

	Medication reduction group (*n* = 282)	Usual care group (*n* = 287)
**Participant characteristics**		
Age (years), mean (SD)	84.6 (3.3)	85.0 (3.5)
Sex (% female)	131 (46.5%)	145 (50.5%)
Body mass index (mean [SD]; kg/m^2^) (n = 534)	27.2 (4.2)	28.0 (4.3)
Ethnicity (% white)	278 (98.6%)	278 (96.9%)
Current smoker (%)	3 (1.1%)	5 (1.7%)
Alcohol consumption (% reporting drinking alcohol every week)	98 (34.8%)	108 (37.6%)
Montreal cognitive assessment score^a^ (mean [SD]) (n = 562)	24.4 (3.6)	24.0 (4.1)
EQ-5d-5L index^b^ (mean [SD]) (n = 563)	0.78 (0.17)	0.76 (0.17)
Modified rankin scale^c^ (% score >2 [dependant]) (n = 540)	36 (12.8%)	42 (14.6%)
Electronic frailty index (eFI),^d^ mean (SD)	0.14 (0.07)	0.15 (0.07)
Fit (eFI 0–0.12; %)	121 (42.9%)	109 (38.0%)
Mild (eFI >0.12–0.24; %)	132 (46.8%)	143 (49.8%)
Moderate (eFI >0.24–0.36; %)	27 (9.6%)	32 (11.1%)
Severe (eFI >0.36; %)	2 (0.7%)	3 (1.0%)
Systolic blood pressure (mmHg), mean (SD)	129.4 (13.1)	130.5 (12.3)
Diastolic blood pressure (mmHg), mean (SD)	68.4 (9.1)	70.1 (8.4)
Orthostatic hypotension (%), (*n* = 525)^e^	15 (5.7%)	10 (3.8%)
**Medical history**		
Chronic kidney disease (%)	83 (29.4%)	103 (35.9%)
Cancer (%)	67 (23.8%)	68 (23.7%)
Cardiac disease (%)^f^	61 (21.6%)	61 (21.3%)
Diabetes (%)	48 (17.0%)	53 (18.5%)
Atrial fibrillation (%)	45 (16.0%)	45 (15.7%)
Transient ischemic attack (%)	27 (9.6%)	22 (7.7%)
Stroke (%)	23 (8.2%)	22 (7.7%)
Peripheral vascular disease (%)	6 (2.1%)	9 (3.1%)
Number of morbidities, mean (SD)	5.7 (2.7)	6.0 (2.9)
% ≥2 morbidities (%)	278 (98.6%)	282 (98.3%)
**Medication prescriptions**		
Antihypertensive (%)^g^	282 (100.0%)	287 (100.0%)
ACE inhibitor (%)	139 (49.3%)	128 (44.8%)
Angiotensin II receptor blocker (%)	99 (35.2%)	115 (40.1%)
Calcium channel blockers (%)	199 (70.6%)	191 (66.6%)
Thiazide and related diuretics (%)	109 (38.7%)	111 (38.7%)
Beta-blockers (%)	112 (39.7%)	116 (40.4%)
Alpha-blockers (%)	41 (14.5%)	39 (13.6%)
Other antihypertensives (%)	19 (6.7%)	35 (12.3%)
Statin (%)	97 (34.4%)	92 (32.1%)
Antiplatelet (%)	58 (20.6%)	53 (18.5%)
Total prescribed medications, median (IQR)	4 (3–7)	4 (3–7)

^a^ Score ranges between 0 and 30 with lower scores representing greater impairment. A score of 26 and over is considered to be normal.

^b^ The EQ-5D-5L assesses five aspects of health: mobility, self-care, activities, discomfort, and anxiety/depression. EQ-5D-5L index scores were generated using crosswalk approach which translates the scores for the five EQ-5D-5L items into a single index value. The index value ranges from -0.594 (worse than death) to 1 (full health).

^c^ Modified Rankin scale ranges from 0 (no symptoms) to 5 (severe disability).

^d^ The Electronic Frailty Index has 36 items and is estimated from electronic health records. The index ranges from 0 (fit) to 1 (frail).

^e^ Orthostatic hypotension defined as a decrease in systolic blood pressure of at least 20 mm Hg within 3 min of standing.

^f^ Cardiac disease defined as the presence of myocardial infarction, coronary heart disease, angina or heart failure.

^g^ The sum of percentages for all antihypertensive medication classes may exceed 100%, since participants had to be taking more than one antihypertensive medication to be eligible for the trial.

SD = standard deviation.

The most commonly prescribed medications at baseline were calcium channel blockers (390 participants, 68.5%), ACE inhibitors (267 participants, 46.9%) and beta-blockers (228 participants, 40.1%). Calcium channel blockers were typically prescribed in combination with ACE inhibitors (180 participants, 31.6%), angiotensin II receptor blockers (136 participants, 23.9%) or beta-blockers (131 participants, 23.0%) (Supplementary Table S2). Thiazide and thiazide-like diuretics were the most common drug class chosen by physicians for medication reduction (168 participants, 29.6%; 76.4% of those prescribed thiazide and thiazide-like diuretics) (Table 2). There were no between group differences in the drug classes chosen for medication reduction. Higher dose calcium channel blockers, thiazides and thiazide-like diuretics were more commonly selected for withdrawal than lower dose medications within these classes (higher dose 90–91% vs. low dose 9–10%; Table 3 and Supplementary Table S3). In contrast, low dose beta-blockers were more commonly chosen for withdrawal than higher dose beta-blockers (higher dose 13% vs. low dose 87%; Table 3).

**TABLE 2:** Total proportion of medications prescribed and selected for medication reduction by randomized group.

Drug class	Medications prescribed			Medications selected for withdrawal			
	Total (%)	Intervention (%)	Control (%)	Total (%)	Proportion of total prescribed (%)	Intervention (withdrawal attempted) (%)	Control (withdrawal not attempted) (%)
Calcium channel blocker	390 (68.5%)	199 (70.6%)	191 (66.6%)	131 (23.1%)	33.6	64 (22.8%)	67 (23.4%)
ACE inhibitor	267 (47.0%)	139 (49.3%)	128 (44.8%)	68 (12.0%)	25.5	34 (12.1%)	34 (11.9%)
Angiotensin II receptor blocker	214 (37.7%)	99 (35.2%)	115 (40.1%)	55 (9.7%)	25.7	27 (9.6%)	28 (9.8%)
Thiazide or thiazide-like diuretic	220 (38.8%)	109 (38.8%)	111 (38.8%)	168 (29.6%)	76.4	88 (31.3%)	80 (27.8%)
Beta-blocker	228 (40.1%)	112 (39.7%)	116 (40.6%)	77 (13.6%)	33.8	36 (12.8%)	41 (14.3%)
Alpha-blocker	80 (14.1%)	41 (14.5%)	39 (13.6%)	43 (7.6%)	53.8	22 (7.8%)	21 (7.3%)
Other antihypertensive	54 (9.5%)	19 (6.7%)	35 (12.2%)	25 (4.4%)	46.3	10 (3.6%)	15 (5.2%)

ACE = angiotensin converting enzyme.

**TABLE 3:** Antihypertensive medications chosen for withdrawal at baseline by drug class and medication dose.

Drug	Low dose medication withdrawal subgroup (<DDD)			Higher dose medication withdrawal subgroup (≥DDD)		
	Total (%)	Intervention (withdrawal attempted) (%)	Control (withdrawal not attempted) (%)	Total (%)	Intervention (withdrawal attempted) (%)	Control (withdrawal not attempted) (%)
Calcium channel blockers	13 (9.9%)	9 (6.9%)	4 (3.1%)	118 (90.1%)	55 (42.0%)	63 (48.1%)
ACE inhibitors	18 (26.5%)	11 (16.2%)	7 (10.3%)	50 (73.5%)	23 (33.8%)	27 (39.7%)
Angiotensin II receptor blockers	18 (32.7%)	6 (10.9%)	12 (21.8%)	37 (67.3%)	21 (38.2%)	16 (29.1%)
Thiazide and thiazide-like diuretics	15 (9.1%)	11 (6.7%)	4 (2.4%)	149 (90.9%)	74 (45.1%)	75 (45.7%)
Beta-blockers	66 (86.8%)	29 (38.2%)	37 (48.7%)	10 (13.2%)	6 (7.9%)	4 (5.3%)
Alpha-blockers	19 (44.2%)	10 (23.3%)	9 (20.9%)	24 (55.8%)	12 (27.9%)	12 (27.9%)
Other antihypertensives	22 (73.3%)	7 (23.3%)	15 (50.0%)	8 (26.7%)	4 (13.3%)	4 (13.3%)

ACE = angiotensin converting enzyme; DDD = defined daily dose.

### Association Between Medication Reduction and Outcomes by Drug Class

**FIGURE 2:**
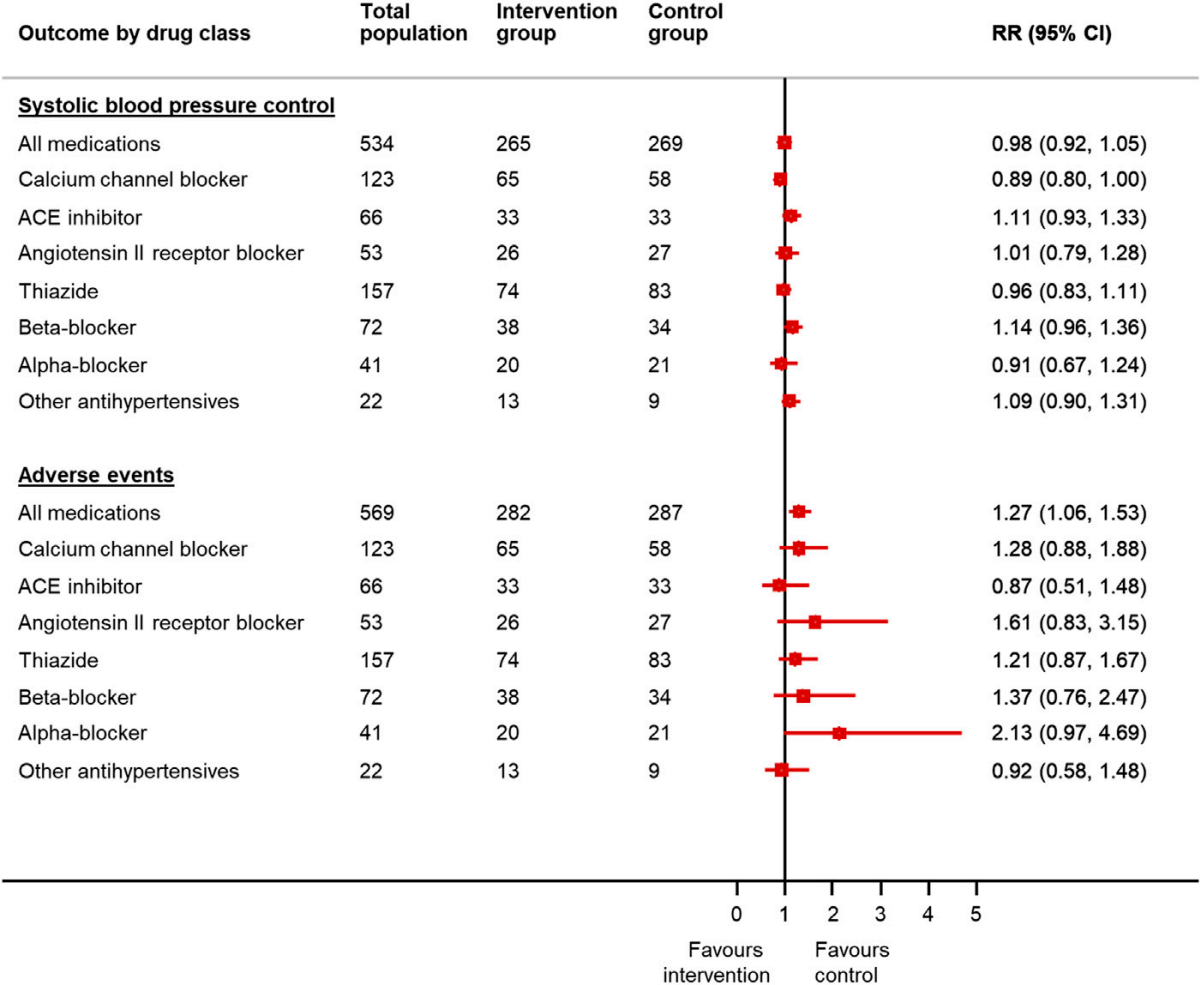
Relative risk of blood pressure control and adverse events in patients reducing antihypertensive medication compared to usual care, by drug class chosen for withdrawal*. *Since the treating physician’s choice of medication to withdraw was made prior to consent and randomization, data were available for all randomized participants, even though only half went on to have the medication withdrawn in the trial. RR = relative risk; CI = confidence interval. Generalized linear mixed model with binomial error and log link, with factors predictive of physician choice of drug to withdraw (see Table 2) and baseline systolic blood pressure, gender, cognitive function (MoCA Score), EQ-5D-5L Index and Searle Frailty Index as fixed effects.

**FIGURE 3:**
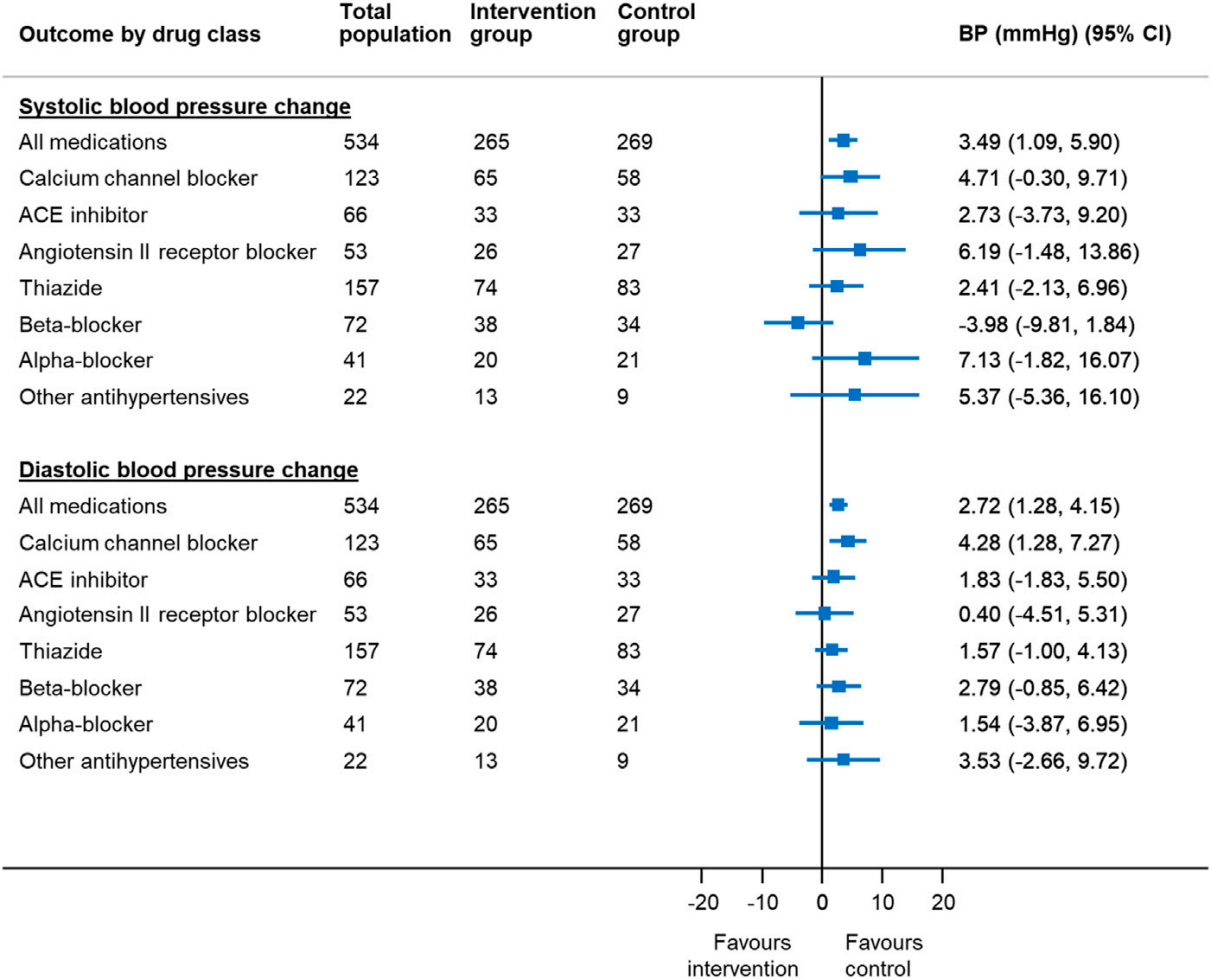
Mean change in blood pressure in patients reducing antihypertensive medication compared to usual care, by drug class chosen for withdrawal* *Since the treating physician’s choice of medication to withdraw was made prior to consent and randomization, data were available for all randomized participants, even though only half went on to have the medication withdrawn in the trial. BP = blood pressure; CI = confidence interval Generalized linear mixed model with binomial error and log link, with factors predictive of physician choice of drug to withdraw (see Table 2) and baseline systolic blood pressure, gender, cognitive function (MoCA Score), EQ-5D-5L Index and Searle Frailty Index as fixed effects and primary care site as a random effect.

After adjusting for factors predictive of drug choice for medication reduction (Supplementary Table S4), participants were less likely to have controlled systolic blood pressure at follow-up if reducing calcium channel blockers (adjusted RR 0.89 95% CI 0.80–0.998) (Figure 2). Withdrawal of calcium channel blockers was also associated with an increase in systolic and diastolic blood pressure (4.7 mm Hg, 95% CI −0.3–9.7 mm Hg [systolic]; 4.3 mm Hg, 95% CI 1.3–7.3 mm Hg [diastolic]) (Figure 3). Withdrawal of beta-blockers was associated with a non-significant reduction in systolic blood pressure (–4.0 mmHg, 95% CI –9.8 to 1.8 mmHg). There was no association between withdrawal of specific drug classes and adverse events (e.g. increased blood pressure, chest pain, infections, ankle swelling, headache and back pain, etc.).

### Association Between Medication Reduction and Outcomes by Medication Dose

**FIGURE 4:**
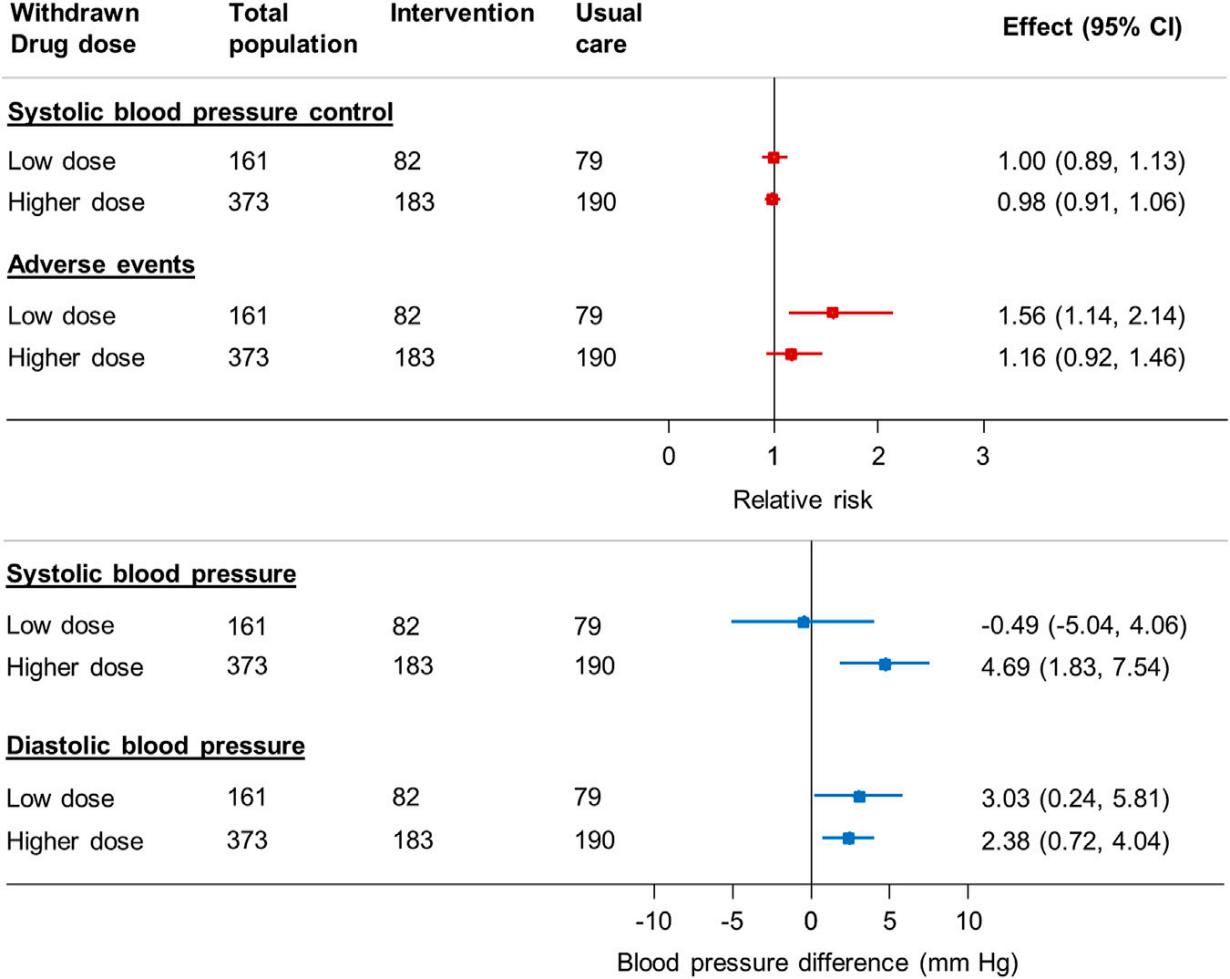
Relative risk of blood pressure control, adverse events and mean change in blood pressure in patients reducing antihypertensive medication compared to usual care, by dose of medication chosen for withdrawal* *Since the treating physician’s choice of medication to withdraw was made prior to consent and randomization, data were available for all randomized participants, even though only half went on to have the medication withdrawn in the trial. BP = blood pressure; CI = confidence interval Generalized linear mixed model with binomial error and log link, with factors predictive of physician choice of drug to withdraw (see table two) and baseline systolic blood pressure, gender, cognitive function (MoCA Score), EQ-5D-5L Index and Searle Frailty Index as fixed effects and primary care site as a random effect.

Withdrawal of higher dose medications was associated with an increase in systolic and diastolic blood pressure (4.7 mm Hg, 95% CI 1.8–7.5 mm Hg [systolic]; 2.4 mm Hg, 95% CI 0.7–4.0 mm Hg [diastolic]) but no difference in blood pressure control (adjusted RR 0.98 95% CI 0.92–1.46) (Figure 4). Withdrawal of low dose medications was not associated with any difference in systolic blood pressure (–0.5 mm Hg, 95% CI –5.0 to 4.1 mmHg) or blood pressure control (adjusted RR 1.00 95% CI 0.89–1.13) between groups. However, withdrawal of low dose medications was associated with an increased risk of adverse events (adjusted RR 1.56 95% CI 1.14–2.14).

### Maintenance of Medication Reduction

All 282 patients randomized to the intervention arm of the trial attempted to withdraw the medication chosen by their primary care physician. Overall, 91 (32.4%) had their medication reintroduced and 101 (35.9%) experienced no increase in systolic blood pressure at 12 weeks follow-up (Supplementary Table S5). The highest proportion of participants maintaining medication reduction and experiencing no increase in systolic blood pressure were those reducing ACE inhibitors (79.4 and 44.1% respectively) and beta-blockers (80.6 and 55.6% respectively). There was no difference in the proportion maintaining medication reduction between those withdrawing higher dose medications and those withdrawing low dose medications (higher dose 66.3% vs. low dose 70.4%).

## Discussion

The OPTiMISE trial (Sheppard et al., 2020c) found that one antihypertensive medication could be withdrawn in the majority of participants without substantial change in blood pressure control at 12 weeks follow-up. This post-hoc exploratory analysis found some evidence to suggest that beta-blockers in particular, especially those prescribed at low doses, may be withdrawn with little or no increase in blood pressure. This makes them a potential target for deprescribing in older patients with no other compelling indication for therapy. Withdrawal of higher dose calcium channel blockers was associated with a reduced likelihood of blood pressure control at follow-up, despite these medications being less likely to be selected for medication reduction in participants with higher baseline blood pressures. This supports recommendations for the use of calcium channel blockers as a first line therapy for hypertension in older patients and suggests these might be avoided as a target for deprescribing. These analyses were exploratory in nature and further larger, appropriately powered studies are needed to confirm these findings in older patients with multi-morbidity and polypharmacy.

### Strengths and Limitations

This is the first analysis of medication reduction by antihypertensive drug class and medication dose using data from a randomized controlled trial (Sheppard et al., 2020c). The trial was successful in recruiting a mildly frail population with multi-morbidity and polypharmacy, representative of older patients attending primary care in England. This was a post-hoc, exploratory analysis, which may have been underpowered to show definitive associations between drug classes, particularly for alpha-blockers and ‘other’ antihypertensives that were chosen for withdrawal in less than 50 trial participants. Since multiple statistical analyses were conducted, the significant associations between withdrawal of calcium channel blockers, higher dose medications and blood pressure at follow-up may have been observed by chance and so these results should be interpreted with caution.

Although follow-up was achieved in 93.8% of participants, the period of follow-up was short, and so it was not possible to examine clinical endpoints such as hospitalization, cardiovascular disease or death at this stage, though the cohort will be followed up. In addition, although routine prescription of beta-blockers is often accompanied by monitoring of heart rate, we did not collect this or related outcomes (e.g. development of atrial fibrillation) during follow-up, precluding any analyses of these outcomes.

### Comparison With Previous Literature

Previous trials of antihypertensive medication reduction have only attempted medication reduction in up to two thirds of participants (Moonen et al., 2015; Gulla et al., 2018; Luymes et al., 2018), had smaller sample sizes (Moonen et al., 2015; Gulla et al., 2018), examined younger populations (i.e. aged less than 80 years) (Luymes et al., 2018) and lacked comparisons with a control group to determine the effect of deprescribing on outcomes (Gulla et al., 2018). This is the first analysis of any previous trial examining deprescribing by drug class and medication dose, providing preliminary data which should be explored in future appropriately powered studies. This might involve attempting to pool data from previous trials (Moonen et al., 2015; Gulla et al., 2018; Luymes et al., 2018) to increase the power to detect effects.

### Implications for Clinical Practice

Physicians participating in the OPTiMISE trial (Sheppard et al., 2020c) were given the freedom to choose which medication should be withdrawn if participants were randomized to the intervention arm of the trial. Advice was given in the form of a medication reduction algorithm which recommended reducing medications in reverse of the C + A + D NICE treatment algorithm (National Guideline Centre, 2019) i.e.; if a participant was prescribed three antihypertensive medications including a thiazide or thiazide-like diuretic, this was recommended to be removed instead of a renin-angiotensin system medication or a calcium channel blocker. In the present analysis, 3 out of 4 patients prescribed a thiazide and thiazide-like diuretic had this medication chosen for withdrawal and increasing number of antihypertensive medications prescribed was one of the strongest predictors of this choice, suggesting that the medication reduction algorithm was followed as suggested.

Calcium channel blockers were less likely to be chosen for medication reduction in patients with higher baseline systolic blood pressure and despite this, withdrawal of these medications was associated with a higher likelihood of uncontrolled blood pressure at follow-up. One explanation for this might be that these medications were predominantly prescribed at higher doses, where the blood pressure lowering effect might be expected to be greater. There is also evidence to suggest that calcium channel blockers are more effective in older individuals, leading to recommendations in clinical guidelines that these should be used as a first line therapy (Williams et al., 2018; National Guideline Centre, 2019). These findings reinforce recommendations in the medication withdrawal algorithm used in the trial, which suggested that these medications should be considered last for medication withdrawal.

The proportion of patients prescribed beta-blockers at baseline was relatively high, particularly since patients with a history of heart failure due to left ventricular dysfunction were excluded (Sheppard et al., 2018). Given that many participants had been diagnosed with hypertension for many years, it is possible that beta-blockers were originally prescribed at a time when they were recommended as a first line treatment for hypertension (Williams et al., 2004). Although subsequent guidelines have changed this recommendation (Mayor, 2006), many patients could have remained on the same treatment as originally prescribed.

These data show that a high proportion of patients withdrawing beta-blockers maintained medication reduction at follow-up and that withdrawal of such medications may be associated with no change or even a reduction in systolic blood pressure. Beta-blockers were more likely to be prescribed at lower doses for patients enrolled into the trial, and selected for medication reduction if participants were prescribed a higher number of antihypertensive medications at baseline. Since polypharmacy is associated with reduced adherence to medications (Smaje et al., 2018), it is possible that withdrawal of beta-blockers may have increased an individual’s adherence to their remaining medications causing blood pressure to be reduced at follow-up, although one might expect this to also be the case for withdrawal of any medication in patients taking multiple antihypertensives.

While withdrawing low-dose beta-blockers with no resulting increase in blood pressure maybe an appealing strategy for physicians, it is important to note that beta-blockers have other cardio-protective properties and may be indicated for other reasons beyond hypertension, such as ischemic heart disease, tachycardia and heart failure with reduced ejection fraction. There was also some evidence to suggest that withdrawal of low dose medications resulted in an increase in adverse events, although these varied widely in terms of severity (e.g. increased blood pressure, chest pain, infections, ankle swelling, headache and back pain). Only 23 participants (13 in the medication reduction group and 10 in the usual care group) experienced a serious adverse event resulting in hospitalization during the trial (Sheppard et al., 2020c). Until studies with long-term follow-up are conducted, it is difficult to draw firm conclusions regarding the choice of medication to withdraw first as part of a deprescribing intervention.

## Conclusion

This exploratory analysis found some evidence to suggest that withdrawal of higher dose calcium channel blockers should be avoided if the goal is to maintain blood pressure control. However, low dose beta-blockers may be removed with little impact on blood pressure at follow-up. More appropriately powered studies are needed to determine whether withdrawal of certain drug classes and/or doses are preferable over others in older patients with multi-morbidity and polypharmacy.

## OPTiMISE Investigators

Julie Allen, Sue Jowett, Jill Mollison, Eleanor Temple, Carl Heneghan, Ly-Mee Yu, Marney Williams, James P. Sheppard, Mark Lown, Jenni Burt, Gary A. Ford, F. D. Richard Hobbs, Paul Little, Jonathan Mant, Rupert A. Payne, Richard J. McManus.

## Data Availability

The raw data supporting the conclusion of this article will be made available by the authors, without undue reservation.
