# Supplementary material for: Blood Pressure Changes Following Antihypertensive Medication Reduction, by Drug Class and Dose Chosen for Withdrawal: Exploratory Analysis of Data From the OPTiMISE Trial
Source: Front Pharmacol. 2021 Apr 20;12:619088. doi: 10.3389/fphar.2021.619088 (PMC8093867; doi:10.3389/fphar.2021.619088)
Supplement: Supplementary file 1 [file datasheet1.docx]

**Blood pressure changes following antihypertensive medication reduction, by drug class and dose chosen for withdrawal: Exploratory analysis of data from the OPTiMISE trial**

**Supplementary material**

James P Sheppard, Mark Lown, Jenni Burt, Gary A Ford, FD Richard Hobbs, Paul Little, Jonathan Mant, Rupert A Payne, Richard J McManus *on behalf of the OPTiMISE Investigators*

**Contents**

1. **eTable 1.** Comparison of the characteristics of all patients aged ≥80 years at 24 general practices recruiting to the trial with those of participants enrolled into the trial.
2. **eTable 2.** Combinations of antihypertensive drugs prescribed at baseline
3. **eTable 3.** Details of antihypertensive medications chosen for withdrawal at baseline
4. **eTable 4.** Predictors of medication reduction drug choice by general practitioners at the beginning of the trial (n=569)
5. **eTable 5.** Proportion of patients in the intervention group maintaining medication reduction and experiencing no increase in systolic blood pressure by drug class

**eTable 1.** Comparison of the characteristics of all patients aged ≥80 years at 24 general practices recruiting to the trial with those of participants enrolled into the trial.

|  | Total  population | Eligible population | Recruited population |
| --- | --- | --- | --- |
| Patient characteristics |  |  |  |
| Total population | 15,376 | 3,940 | 569 |
| Age (years) | 85.5 | 84.8 | 84.8 |
| Sex (% female) | 58.8% | 58.0% | 51.5% |
| Systolic blood pressure (mm Hg) | 132.7 | 130.8 | 130.0 |
| Diastolic blood pressure (mm Hg) | 72.7 | 71.0 | 69.3 |
| Body mass index (kg/m^2^) | 26.4 | 27.7 | 27.6 |
| Electronic frailty index (median) | 0.12 | 0.13 | 0.14 |
| Medical history |  |  |  |
| Hypertension | 61.8% | 89.4% | 96.1% |
| Cardiovascular disease | 35.8% | 42.4% | 30.4% |
| Chronic kidney disease | 28.2% | 36.8% | 32.7% |
| Diabetes | 16.5% | 23.0% | 17.8% |
| Stroke/transient ischemic attack | 14.6% | 13.0% | 7.9% |
| Myocardial infarction | 7.3% | 10.1% | 7.0% |
| Prescribed medications |  |  |  |
| Antihypertensive | 64.2% | 100.0% | 100.0% |
| Statin | 42.2% | 61.0% | 33.2% |
| Antiplatelet | 25.4% | 31.8% | 19.5% |
| Mean number of antihypertensives prescribed | 1.1 | 2.4 | 2.5 |

Total and eligible populations estimated using data from general practices using the EMIS electronic health record system. These anonymized data were extracted from the electronic health records of all individuals aged 80 and older and registered at 24 general practices hosting the OPTiMISE trial. Hypertension is defined in this table as a coded diagnosis on the electronic health records, although in the context of trial eligibility, patients were considered hypertensive if they had a coded diagnosis or were taking antihypertensive medication to lower blood pressure.

**eTable 2.** Combinations of antihypertensive drugs prescribed at baseline

| Drug class | ACE inhibitor | Angiotensin II receptor blocker | Thiazide or thiazide-like diuretic | Beta-blocker | Alpha-blocker | Other antihypertensive |
| --- | --- | --- | --- | --- | --- | --- |
| Calcium channel blocker | 180 | 136 | 121 | 131 | 40 | 23 |
| ACE inhibitor | | 4 | 100 | 97 | 29 | 16 |
| Angiotensin II receptor blocker | | | 72 | 81 | 35 | 26 |
| Thiazide or thiazide-like diuretic | | | | 71 | 22 | 10 |
| Beta-blocker | | | | | 22 | 30 |
| Alpha-blocker | | | | | | 6 |

**eTable 3.** Details of antihypertensive medications chosen for withdrawal at baseline

| Drug | Defined daily dose (DDD) | Low dose medication withdrawal subgroup (<DDD) | | | | Higher dose medication withdrawal subgroup (≥DDD) | | | | |
| --- | --- | --- | --- | --- | --- | --- | --- | --- | --- | --- |
|  |  | **Intervention group** | | **Usual care group** | | **Intervention group** | | **Usual care group** | | |
|  |  | **Total** | **%** | **Total** | **%** | **Total** | **%** | | **Total** | **%** |
| Total population |  | 88 |  | 86 |  | 194 |  | | 201 |  |
| ACE inhibitors | | **11** | **12.5%** | **7** | **8.1%** | **23** | **11.9%** | | **27** | **13.4%** |
| Ramipril | 2.5 mg | 5 | 5.7% | 2 | 2.3% | 16 | 8.2% | | 17 | 8.5% |
| Lisinopril | 10 mg | 3 | 3.4% | 3 | 3.5% | 4 | 2.1% | | 9 | 4.5% |
| Perindopril | 4 mg | 2 | 2.3% | 1 | 1.2% | 1 | 0.5% | | 0 | 0.0% |
| Enalapril | 12 mg | 0 | 0.0% | 1 | 1.2% | 2 | 1.0% | | 1 | 0.5% |
| Fosinopril | 15 mg | 1 | 1.1% | 0 | 0.0% | 0 | 0.0% | | 0 | 0.0% |
| Angiotensin II receptor blockers | | **6** | **6.8%** | **12** | **14.0%** | **21** | **10.8%** | | **16** | **8.0%** |
| Losartan | 50 mg | 1 | 1.1% | 8 | 9.3% | 4 | 2.1% | | 9 | 4.5% |
| Candesartan | 8 mg | 5 | 5.7% | 2 | 2.3% | 8 | 4.1% | | 4 | 2.0% |
| Irbesartan | 150 mg | 0 | 0.0% | 0 | 0.0% | 3 | 1.5% | | 0 | 0.0% |
| Telmisartan | 40 mg | 0 | 0.0% | 0 | 0.0% | 1 | 0.5% | | 2 | 1.0% |
| Eprosartan | 600 mg | 0 | 0.0% | 1 | 1.2% | 3 | 1.5% | | 0 | 0.0% |
| Olmesartan | 20 mg | 0 | 0.0% | 1 | 1.2% | 1 | 0.5% | | 1 | 0.5% |
| Valsartan | 80 mg | 0 | 0.0% | 0 | 0.0% | 1 | 0.5% | | 0 | 0.0% |
| Calcium channel blockers | | **9** | **10.2%** | **4** | **4.7%** | **55** | **28.4%** | | **63** | **31.3%** |
| Amlodipine | 5 mg | 1 | 1.1% | 0 | 0.0% | 45 | 23.2% | | 41 | 20.4% |
| Felodipine | 5 mg | 5 | 5.7% | 1 | 1.2% | 6 | 3.1% | | 8 | 4.0% |
| Lercanidipine | 10 mg | 0 | 0.0% | 0 | 0.0% | 4 | 2.1% | | 11 | 5.5% |
| Nifedipine | 30 mg | 0 | 0.0% | 3 | 3.5% | 0 | 0.0% | | 2 | 1.0% |
| Lacidipine | 4 mg | 0 | 0.0% | 0 | 0.0% | 0 | 0.0% | | 1 | 0.5% |
| Diltiazem | 240 mg | 3 | 3.4% | 0 | 0.0% | 0 | 0.0% | | 0 | 0.0% |
| Thiazide and thiazide-like diuretics | | **11** | **12.5%** | **4** | **4.7%** | **74** | **38.1%** | | **75** | **37.3%** |
| Bendroflumethiazide | 2.5 mg | 1 | 1.1% | 0 | 0.0% | 65 | 33.5% | | 65 | 32.3% |
| Indapamide | 2.5 mg | 7 | 8.0% | 4 | 4.7% | 9 | 4.6% | | 10 | 5.0% |
| Hydrochlorothiazide | 25 mg | 2 | 2.3% | 0 | 0.0% | 0 | 0.0% | | 0 | 0.0% |
| Chlortalidone | 25 mg | 1 | 1.1% | 0 | 0.0% | 0 | 0.0% | | 0 | 0.0% |
| Beta-blockers | | **29** | **33.0%** | **37** | **43.0%** | **6** | **3.1%** | | **4** | **2.0%** |
| Atenolol | 75 mg | 15 | 17.0% | 18 | 20.9% | 6 | 3.1% | | 2 | 1.0% |
| Metoprolol | 150 mg | 0 | 0.0% | 1 | 1.2% | 0 | 0.0% | | 0 | 0.0% |
| Bisoprolol | 10 mg | 14 | 15.9% | 15 | 17.4% | 0 | 0.0% | | 1 | 0.5% |
| Sotalol | 160 mg | 0 | 0.0% | 0 | 0.0% | 0 | 0.0% | | 1 | 0.5% |
| Propranolol | 160 mg | 0 | 0.0% | 2 | 2.3% | 0 | 0.0% | | 0 | 0.0% |
| Nebivolol | 5 mg | 0 | 0.0% | 1 | 1.2% | 0 | 0.0% | | 0 | 0.0% |
| Alpha-blockers | | **10** | **11.4%** | **9** | **10.5%** | **12** | **6.2%** | | **12** | **6.0%** |
| Doxazocin | 4 mg | 9 | 10.2% | 9 | 10.5% | 11 | 5.7% | | 11 | 5.5% |
| Tamsulosin | 0.4 mg | 0 | 0.0% | 0 | 0.0% | 1 | 0.5% | | 1 | 0.5% |
| Prazosin | 5 mg | 1 | 1.1% | 0 | 0.0% | 0 | 0.0% | | 0 | 0.0% |
| Other antihypertensives | | **7** | **8.0%** | **15** | **17.4%** | **4** | **2.1%** | | **4** | **2.0%** |
| Amiloride | 10 mg | 0 | 0.0% | 2 | 2.3% | 0 | 0.0% | | 0 | 0.0% |
| Spironolactone | 10 mg | 1 | 1.1% | 3 | 3.5% | 0 | 0.0% | | 0 | 0.0% |
| Hydralazine | 100 mg | 0 | 0.0% | 1 | 1.2% | 0 | 0.0% | | 0 | 0.0% |
| Moxonidine | 0.3 mg | 2 | 2.3% | 2 | 2.3% | 1 | 0.5% | | 0 | 0.0% |
| Furosemide | 40 mg | 4 | 4.5% | 7 | 8.1% | 2 | 1.0% | | 3 | 1.5% |
| Bumetanide | 1 mg | 0 | 0.0% | 0 | 0.0% | 1 | 0.5% | | 1 | 0.5% |

ACE=Angiotensin converting enzyme

**eTable 4.** Predictors of medication reduction drug choice by general practitioners at the beginning of the trial (n=569)

| Predictor | CCB  OR (95% CI) | ACE inhibitor  OR (95% CI) | ARB  OR (95% CI) | Thiazides  OR (95% CI) | Beta blocker  OR (95% CI) | Alpha blocker  OR (95% CI) | Other  OR (95% CI) |
| --- | --- | --- | --- | --- | --- | --- | --- |
| Age  (per 1 year increment) | 1.06  (1.01-1.13) | 1.00  (0.92-1.08) | 0.97  (0.90-1.06) | 0.99  (0.94-1.05) | 0.95  (0.88-1.03) | 0.995  (0.91-1.09) | 0.96  (0.85-1.09) |
| Sex  (ref category: female) | 1.62  (1.07-2.47) | 1.05  (0.61-1.80) | 0.85  (0.47-1.52) | 0.95  (0.65-1.39) | 0.60  (0.36-0.99) | 1.40  (0.73-2.69) | 0.60  (0.26-1.39) |
| Systolic blood pressure  (per 1 mmHg increment) | 0.98  (0.96-0.99) | 1.04  (1.01-1.06) | 0.996  (0.97-1.02) | 1.01  (0.99-1.03) | 1.01  (0.99-1.03) | 0.99  (0.97-1.01) | 0.99  (0.96-1.02) |
| Number AHT medications  (per 1 medication increment) | 0.76  (0.55-1.05) | 0.24  (0.13-0.45) | 0.37  (0.21-0.65) | 1.56  (1.18-2.06) | 1.47  (1.03-2.09) | 1.16  (0.73-1.85) | 3.00  (1.76-5.11) |
| Number of medications  (per 1 medication increment) | 1.05  (0.98-1.11) | 1.02  (0.94-1.12) | 1.06  (0.97-1.16) | 0.91  (0.85-0.97) | 0.99  (0.91-1.07) | 0.99  (0.88-1.09) | 1.08  (0.97-1.21) |
| Cardiovascular disease  (ref category: no disease) | 1.44  (0.87-2.39) | 1.05  (0.49-2.24) | 1.47  (0.72-3.03) | 0.67  (0.40-1.12) | 1.12  (0.58-2.14) | 0.54  (0.21-1.41) | 0.99  (0.36-2.71) |
| Number of morbidities  (per 1 condition increment) | 0.94  (0.87-1.07) | 0.97  (0.81-1.16) | 1.00  (0.84-1.20) | 1.02  (0.90-1.15) | 1.06  (0.90-1.23) | 0.96  (0.78-1.18) | 1.18  (0.93-1.51) |
| Electronic frailty index  (per 0.1 increment) | 1.35  (0.79-2.31) | 0.94  (0.46-1.91) | 1.04  (0.50-2.19) | 0.97  (0.59-1.60) | 0.73  (0.38-1.40) | 1.23  (0.52-2.89) | 0.54  (0.18-1.55) |

Shaded cells indicate those predictors with a significant association with choice of antihypertensive for medication reduction; CCB=calcium channel blocker; ACE=angiotensin converting enzyme; ARB=angiotensin II receptor blocker; AHT=antihypertensive; OR=odds ratio; CI=confidence interval.

The number of participants in whom each drug class was selected for withdrawal is given eTable 3.

**eTable 5.** Proportion of patients in the intervention group maintaining medication reduction and experiencing no increase in systolic blood pressure by drug class

| Total | Total population | Maintenance of medication reduction | % | No increase in SBP | % |
| --- | --- | --- | --- | --- | --- |
| Overall | 281 | 190 | 67.6% | 101 | 35.9% |
| Calcium channel blocker | 64 | 33 | 51.6% | 17 | 26.6% |
| ACE inhibitor | 34 | 27 | 79.4% | 15 | 44.1% |
| Angiotensin II receptor blocker | 27 | 18 | 66.7% | 8 | 29.6% |
| Thiazide | 88 | 60 | 68.2% | 33 | 37.5% |
| Beta-blocker | 36 | 29 | 80.6% | 20 | 55.6% |
| Alpha-blocker | 22 | 15 | 68.2% | 6 | 27.3% |
| Other antihypertensive | 10 | 8 | 80.0% | 2 | 20.0% |

SBP=systolic blood pressure; ACE=angiotensin converting enzyme
